# Welfare first: transforming harm reduction at UK festivals

**DOI:** 10.1186/s12954-025-01184-1

**Published:** 2025-03-22

**Authors:** Joseph Janes

**Affiliations:** Swansea University, Swansea, UK

## Abstract

**Background:**

A welfare-first approach to harm reduction at UK festivals is emerging as a critical strategy for enhancing festival safety. In particular, the implementation of anonymous, non-punitive drug-checking services is posited as essential for reducing drug-related harm by enabling informed decision-making. This empirical study examines the limitations of punitive drug policies and the associated risks to public health and explores the potential benefits of decriminalisation in fostering safer festival environments.

**Methods:**

The study employed qualitative methodologies, including semi-structured interviews and questionnaires with festival attendees at three major UK festivals. This empirical data was supplemented by a review of recent studies (Ivers et al. in Ir J Med Sci 191(4):1701–1710, 2022. 10.1007/s11845-021-02765-2; Palmer Maynard in Harm Reduc J 19(1):81, 2022. 10.1186/s12954-022-00662-0; Cooney and Measham. in Drug Sci Policy Law 9, 2023. 10.1177/20503245231211444) and relevant policy documents, in order to evaluate current harm reduction practices and identify key barriers, such as stigma, social control, and criminalisation.

**Results:**

Analysis revealed that integrated harm reduction measures, comprising drug-checking services, welfare support, and early intervention initiatives, significantly enhance safety by empowering individuals with timely, accurate substance information. A majority of participants expressed a clear preference for drug-checking services, underscoring their willingness to engage when these services are provided in a supportive, non-punitive environment. However, persistent challenges related to punitive drug policies and gaps in public education about harm reduction continue to impede optimal service delivery. Evidence further suggests that a shift towards decriminalisation and welfare-based approaches could mitigate these risks and foster more trusting engagement with harm reduction initiatives.

**Conclusions:**

The findings indicate that prioritising welfare-first harm reduction strategies, particularly the implementation of anonymous drug-checking services, can create safer festival environments and inform broader public health policies. The study underscores the need for policy reforms that move away from punitive approaches, suggesting that festival-based interventions can serve as scalable models for reducing drug-related harm across diverse community settings.

## Background

Harm reduction (HR) strategies and welfare-based principles play a critical role in enhancing safety at festivals. Among these, drug-checking services provide an essential public health function, enabling individuals to make informed decisions about substance use. This study draws on empirical data from three major UK festivals to assess the effectiveness of HR initiatives and their potential for broader application. Situating the analysis within criminology, harm reduction, and stigma, the paper highlights how these approaches can reduce drug-related harm and challenge punitive drug policies.

UK drug policies have traditionally emphasised abstinence and criminalisation, yet there is a growing recognition of harm reduction as a more effective strategy. This research underscores the need to move beyond punitive approaches and embrace supportive, evidence-based policies that prioritise public health. Drawing on data from 50 participants, the study explores key themes, including social control, policing, and early intervention, to demonstrate how criminalisation exacerbates risks for festival attendees. The findings indicate that welfare-based strategies not only promote safety, but also offer broader insights into policing and drug policy reform.

The development of drug-checking as a harm reduction intervention has positioned it as a legally, politically, and commercially sensitive issue. The implementation and effectiveness of drug-checking services are influenced by multiple factors, including the demographics of service users, the context in which testing occurs, the analytical techniques employed, the speed of result dissemination, the scope of the intervention, and the engagement of key stakeholders. Additionally, legal frameworks, funding structures, and staff training play a critical role in shaping the accessibility and impact of these services [5].

The origins of drug-checking date back to the 1990s, when the Netherlands’ Ministry of Health established the first state-funded drug-checking system, the Drug Information and Monitoring System (DIMS) [61]. DIMS was designed to safeguard public health, reduce drug-related harms, and generate empirical data to inform both policy and harm reduction initiatives. Its public health impact is evident in cases such as the 2008 mass warning issued in the Netherlands and Belgium regarding lethal "pink pills" containing 170 mg of paramethoxymethamphetamine (PMMA). In contrast, the absence of drug-checking services in the UK at the time contributed to four fatalities linked to the same substance.

Beyond its immediate benefits for individuals who use drugs—providing chemical analysis of substances and enabling informed decision-making—drug-checking also serves a broader public health function by offering critical insights into the composition of the illicit drug market. This surveillance capacity strengthens harm reduction efforts by identifying emerging trends, contaminants, and adulterants, thereby facilitating timely interventions to mitigate associated risks [39].

The legal landscape surrounding drug checking in the UK remains complex. While organisations such as The Loop operate in some capacity, legal and policy constraints continue to limit the availability of these services. This paper situates its argument within this context, highlighting the need for clearer legal pathways to support harm reduction initiatives. Recent studies [13, 34, 54] have documented the prevalence of drug-related deaths at UK festivals, with an estimated five to six fatalities occurring annually. Between 2017 and 2023, 32 potential drug-related deaths (DRDs) were recorded at festivals, 18 of which were confirmed [13], including cases involving individuals under 18. Given that festival drug use is significantly higher than in the general population, up to 87% of attendees have tried illegal substances compared to 36% of young adults more broadly [44], there is an urgent need for harm reduction services to be implemented at festivals.

Beyond drug-checking services, broader HR measures such as substance misuse education (SME) are necessary to support young people in making informed choices. However, much of the existing research in this area is outdated, necessitating renewed efforts to understand young people’s knowledge of substances and access to emergency services. Including individuals with lived experience of drug use in HR initiatives is crucial to designing effective interventions [60].

Harm reduction is fundamentally grounded in human rights and justice, emphasising positive change without coercion, discrimination, or mandatory abstinence (HRI, 2022). Conducted in collaboration with iThink Festival Welfare Services, this study assesses the need for expanded HR strategies at UK festivals, advocating for evidence-based, welfare-driven approaches that prioritise safety and well-being. The research also considers the potential for HR strategies to extend beyond festivals, offering insights into public health interventions in areas such as sexual health and skin cancer prevention. By addressing these issues, this paper contributes to the broader discourse on harm reduction and its role in contemporary drug policy reform.

In discussions of festival drug services, it is crucial to distinguish between publicly accessible drug checking and non-public drug testing, as these terms are often conflated. Public drug checking refers to harm reduction services where individuals can voluntarily submit substances for chemical analysis and receive tailored harm reduction advice. These services operate in various international contexts but are very rarely permitted at UK festivals, with the exception of The Loop. In contrast, non-public drug testing occurs behind the scenes, often carried out by event organisers or medical teams to assess the composition of substances circulating onsite [19], an example of this would be WEDINOS, a harm reduction project, providing an anonymous service of drug checking, by submitting a sample to WEDINOS, you surrender the illicit substances in your possession (WEDINOS, 2024).

Events such as Glastonbury and Leeds Festival have historically conducted non-public testing, where results inform harm reduction messaging but are not directly available to individuals using drugs. The distinction is critical because non-public testing lacks the individualised feedback loop that public drug checking provides, potentially limiting its direct harm reduction impact [67]. This paper refers to publicly accessible drug checking.

### Literature review

This article addresses a significant gap in harm reduction strategies and drug-checking services at UK festivals, offering a robust analysis grounded in empirical data from a broad sample of festival attendees. It challenges traditional abstinence-only policies, advocating for decriminalisation and welfare-based approaches to reduce harm and stigma associated with drug use.

### Criminalisation and harm

Current models of criminalisation in the UK instil fear and increase harm among festival attendees by creating a climate of surveillance and punishment [58]. This approach often deters individuals from seeking medical help in emergencies due to the potential legal repercussions associated with drug use [68]. Reluctance to seek help can result in dangerous situations where individuals experience harm due to the absence of immediate medical intervention. Research indicates that stigma and discrimination associated with drug use have detrimental effects on both mental and physical health [1, 46]. Moreover, the heavy emphasis on law enforcement at festivals, such as stop-and-search practices, fosters distrust and discourages safer behaviours like accessing drug-checking services [28, 58].

Criminalisation also exacerbates unsafe practices such as secretive or rushed drug consumption to avoid detection, increasing the risk of overdose and other health complications (UNODC 2019). A particularly concerning trend is the practice of preloading—consuming large quantities of substances before entering festival grounds to avoid being caught with illicit substances. This behaviour, prevalent among festival attendees, has been associated with heightened risks of overdose and other adverse health outcomes [52, 65]. Fear of legal consequences further discourages individuals from seeking help during emergencies, leading to tragic outcomes.

Examples from other jurisdictions demonstrate the potential benefits of alternative approaches. Legal amnesty measures, such as the Samaritan's Law in some regions, provide protection for individuals seeking emergency assistance in drug-related incidents. Implementing similar policies in the UK could reduce the harms associated with substance use at festivals and encourage attendees to seek help without fear of legal repercussions (Drug Policy Alliance, 2018).

### Music festivals and drug testing

The UK’s approach to drug policy and harm reduction at festivals has evolved significantly over the past few decades. Since the 1980s, harm reduction strategies have been subject to policy shifts between public health and punitive approaches [16, 59]. While the UK historically embraced harm reduction in response to the HIV/AIDS crisis, more recent policy directions have emphasised recovery and abstinence-based models [45], often sidelining harm reduction interventions.

Festival drug-checking services in the UK have operated in a legal grey area, influenced by police cooperation and festival organisers’ willingness to engage with harm reduction initiatives. The first publicly accessible drug-checking pilots were introduced in the mid-2010s, with The Loop conducting on-site testing at events such as Secret Garden Party and Kendal Calling. However, these initiatives ceased following Home Office interventions in 2023, highlighting the fragility of harm reduction services within restrictive legal frameworks. Understanding this policy context is essential for evaluating the feasibility of future harm reduction strategies at UK festivals.

Music festivals have become an increasingly popular cultural phenomenon, particularly among young people [51]. In 2019, an estimated 26% of British young people and adults attended a music festival [56]. However, festivals are often environments where atypical drug use occurs, including increased consumption and experimentation, as well as polydrug use [26, 63]. The UK has one of the highest rates of drug-related fatalities at festivals in Europe, with at least 14 young people dying from drug use at music festivals in England since 2017 [35].

Despite these alarming statistics, UK drug policy remains predominantly punitive, focusing on abstinence rather than harm reduction. This stance contrasts with evidence from other countries where harm reduction measures, such as drug-checking services, have been implemented successfully. For example, studies from Australia and Ireland highlight the positive impact of onsite drug testing at festivals, including increased engagement with harm reduction practices and reduced drug-related medical incidents [34, 50]. In the UK, pilot programs have similarly shown promise, with a 12% drop in drug-related medical incidents observed when drug-checking services were available [44].

Drug-testing services allow festival attendees to anonymously submit substances for content analysis, providing valuable information about the composition and associated risks [4]. These services have been shown to reduce harm without increasing drug use or mortality rates [9, 10, 32, 40]. However, their implementation in the UK faces significant challenges, including legal and logistical barriers, as well as public and political resistance [27, 41]. While drug testing has been shown to be effective as a technique for harm reduction and health promotion, the use of such services remains a controversial issue in the United Kingdom. Stemming from concerns about normalising drug use, some argue that such safeguarding measures may promote the normalisation of drug use or create a false sense of security [17, 57, 69].

Harm reduction within festival environments has developed significantly in recent years, with a growing body of research examining its role in mitigating drug-related harms [34]. Large-scale UK festivals often incorporate a combination of medical, welfare, and harm reduction services, yet the structure and accessibility of these interventions vary depending on funding, policy frameworks, and event management priorities [6]. The presence of on-site medical services, welfare provisions, and informal trip-sitting initiatives has become standard at many major festivals, with some events also incorporating non-public drug testing—a behind-the-scenes process where substances found on-site or confiscated by security are tested to inform harm reduction alerts [43]. However, public drug checking, where attendees could voluntarily submit substances for chemical analysis, was trialled at festivals from 2016 to 2022 but was halted due to regulatory barriers [11]. In 2023, UK festival drug testing providers were told by the Home Office they have to apply for a Controlled Drugs Licence, which costs more than £3,000 and takes three months to process [29, 64]. This sudden regulatory shift created significant financial and logistical barriers, making it nearly impossible for festival organisers and harm reduction organisations to continue offering drug-checking services.

The enforcement of licensing requirements faced strong opposition from harm reduction advocates, public health experts, and festival organisers, who argued that it undermined years of progress in reducing drug-related harm at festivals [62]. The Digital, Culture, Media and Sport Committee also urged the Government to remove legal barriers to drug-checking services, emphasising their role in providing life-saving information to festivalgoers [30]. Unlike in several European countries, where drug-checking services receive government support, the UK's restrictive regulatory framework has significantly limited access to such harm reduction measures. As a result, festival attendees now have fewer options for ensuring the safety of substances, potentially heightening the risks associated with illicit drug use. This policy shift highlights broader structural challenges in accessing public drug-checking services, both within festival settings and across wider community contexts.

This policy shift highlights broader structural challenges in accessing public drug-checking services, both within festival settings and across wider community contexts. These challenges also bring into focus the differing landscapes of harm reduction within UK festivals and urban nightlife settings—an important yet often overlooked distinction in the literature. While festivals typically offer more structured harm reduction initiatives, including dedicated welfare spaces and trip-sitting services, similar provisions are largely absent in urban nightlife environments. Licensing restrictions, heightened police scrutiny, and venue liability concerns create significant barriers to implementing harm reduction strategies in clubs and music venues, raising questions about the disparity in drug policy approaches across different leisure spaces.

As previously noted, harm reduction services are well established at many large UK festivals, where integrated welfare, medical, and harm reduction interventions are deployed to mitigate drug-related risks [26, 43]. In contrast, urban nightlife settings, such as clubs and gig venues, face significant obstacles to implementing similar measures due to licensing restrictions, stricter police oversight, and limited funding [48]. This disparity raises important ethical questions about whether a two-tier system in drug control is emerging, festival attendees, who are often middle-class and able to afford ticketed entry, benefit from comprehensive harm reduction services, while patrons of urban nightlife venues, typically drawn from more economically diverse backgrounds, experience stricter enforcement and fewer protections [14].

Festivals, particularly those managed by major promoters, operate on private land and enjoy greater autonomy in integrating harm reduction strategies, often functioning as de facto decriminalised zones with minimal police intervention [42]. Research into the marketisation of UK festivals suggests that the provision of harm reduction services is influenced not only by public health priorities but also by commercial interests and financial constraints [58].

### Harm reduction and welfare-based approaches

Festival harm reduction services are not a monolithic entity but rather a collection of specialised provisions with distinct functions, governance, and staffing models [5]. Medical services, often operated by professional paramedics or NHS-affiliated providers, handle life-threatening drug-related incidents and other medical emergencies. Welfare services, staffed by trained volunteers or third-sector organisations, provide a broader spectrum of support, including crisis intervention, mental health first aid, and emotional support for intoxicated attendees [54]. Some events also include dedicated harm reduction teams, such as trip-sitting services for individuals experiencing acute drug-induced distress [54].

Importantly, these services are funded and managed differently across festivals. In the UK, festival organisers typically finance medical and welfare services, leading to variations in quality and availability depending on budget constraints. In contrast, countries such as Portugal and the Netherlands have public health funding for festival harm reduction, facilitating more integrated and standardised approaches [65]. The financial burden on UK festival organisers raises important questions about who should bear responsibility for ensuring festival safety, a debate that remains unresolved within UK drug policy discourse.

Harm reduction prioritises mitigating the negative consequences of substance use over enforcing abstinence. This approach recognises the inevitability of some level of drug use in society and focuses on reducing associated harms to health, society, and the economy [37]. Methods such as needle exchanges, naloxone distribution, and drug-testing services exemplify harm reduction strategies that have proven effective in reducing morbidity and mortality [2, 36]. In the context of music festivals, harm reduction can be complemented by welfare-based approaches that provide basic provisions such as water, sunscreen, and resting spaces, alongside more specialised support for those experiencing substance-related harm [44]. Welfare services not only enhance the festival experience but also serve as educational platforms, increasing attendees' knowledge of safer drug use practices and emergency responses (Harm Reduction Coalition, 2023).

Organisations like iThink Welfare Services exemplify the potential of integrated harm reduction and welfare approaches. Operating at festivals across the UK, iThink offers a range of services, including drug testing, support for individuals experiencing negative substance use effects, and distribution of harm reduction resources such as condoms and sanitary products [33]. These initiatives demonstrate the practical benefits of shifting from punitive to supportive strategies in addressing substance use at festivals. Despite growing evidence supporting harm reduction and welfare-based approaches, there remains a significant gap in research on UK-specific festival contexts, particularly concerning young people's knowledge and use of these services. This study aims to fill this gap by examining the substance use patterns of young festival attendees and their engagement with harm reduction services, contributing to the broader discourse on public health and drug policy reform.

## Methods

This study employed a mixed-methods approach, integrating semi-structured face-to-face interviews with an online questionnaire to comprehensively explore festival attendees' knowledge and understanding of drugs, substances (including alcohol and nicotine), and harm reduction practices. A total of 35 interviews and 15 online questionnaires were completed, allowing for data triangulation that enhanced both the depth and validity of the findings. The research was conducted at three major UK festivals, Glastonbury, Leeds, and Boardmasters to ensure a diverse and contextually relevant participant base.

The decision to focus on festivals was informed by several key factors. Festivals offer a unique setting that combines a controlled environment with dynamic social interactions, providing fertile ground for implementing and evaluating harm reduction strategies. Moreover, festivals benefit from well-established stakeholder relationships and structured service provision, which facilitate more accessible and reliable data collection compared to other nightlife contexts. This concentrated access to a well-defined sample, coupled with the distinctive organisational structures and regulatory frameworks at festivals, underpinned the decision to focus exclusively on these events, thereby providing a clear picture of harm reduction service implementation in a setting where such measures are both critical and more systematically integrated.

### Participant recruitment and demographics

Participants were recruited on-site at festivals using convenience sampling, reflecting the ethnographic tradition of studying behaviours in their natural settings [24]. Additional recruitment occurred online through festival-related forums and social media platforms, broadening the scope of participation beyond those attending in person. The participant group comprised 50 individuals, with an average age of 22 years and a range spanning 18 to 35 years old. Gender representation was relatively balanced, with 52% identifying as female, 46% as male, and 2% as non-binary. In terms of ethnicity, 75% of participants identified as White British, 15% as Black, Asian, or Minority Ethnic (BAME), and 10% as mixed ethnicity.

Participants were recruited through two main channels: (1) on-site engagement at festival welfare tents and harm reduction service points, where they were approached by researchers or self-selected to participate after seeing study information posters; and (2) online recruitment via social media and festival-related forums. Participants engaged in either an interview or questionnaire. This dual-method design ensured the inclusion of diverse perspectives and allowed for robust triangulation of the data.

### Data collection

Data was collected using two complementary methods: semi-structured interviews and an online questionnaire. The semi-structured interviews were conducted face-to-face at the festivals, offering a rich and nuanced understanding of participants' experiences, perceptions, and attitudes. These interviews lasted between 30 and 60 min and followed a flexible guide to explore topics such as drug use perceptions, harm reduction awareness, and attitudes toward festival drug policies. Interviews were conducted in designated welfare spaces within festival sites to ensure privacy and comfort for participants. Audio recordings were made with participant consent and later transcribed for analysis.

The online questionnaire was designed to complement the qualitative data by capturing both quantitative and qualitative information. It included a mix of Likert-scale questions, open-ended responses, and demographic details. The questionnaire was distributed online after the festivals, enabling the research team to gather additional insights and validate themes emerging from the interviews. Participants were directed to the survey through QR codes displayed at festival harm reduction service points, as well as through targeted social media outreach. The survey aimed to assess participants' drug use patterns, awareness of harm reduction strategies, and engagement with festival-based welfare services.

Ethical considerations were paramount in this study. Informed consent was obtained from all participants before they took part in the research. They were fully informed of the study’s purpose, the voluntary nature of their participation, their right to confidentiality, and their ability to withdraw at any time. Ethical approval for the research was secured from Swansea University's Social Sciences and Humanities Ethics Board, adhering to the guidelines set out by the British Sociological Association (2017), which emphasise respect for participants, the avoidance of harm, and the importance of voluntary participation.

### Analytical framework

The data were analysed using Braun and Clarke’s [8] six-phase thematic analysis framework, with iterative adaptations to suit the dataset. Initially, the researcher familiarised themselves with the transcripts and survey responses, noting emerging ideas. An inductive approach was then used to generate initial codes that incorporated both emerging and a priori themes [20], such as harm reduction awareness. Multiple coders independently applied these codes, and discrepancies were reconciled to ensure consistency. Codes were subsequently organised into themes through systematic pattern recognition and refined by cross-referencing with the raw data [47]. Finally, the refined themes were clearly defined and integrated into the final report with illustrative quotes, ensuring that the analysis robustly captured the complexity of participants’ experiences.

### Research objectives

The overarching aim of this study was to reduce harm and save lives by addressing stigma, enhancing education about drug use, and advocating for harm reduction practices at UK festivals. A central focus of the research was the identification of systemic issues that inhibit harm reduction measures, particularly in festival contexts, where proactive policies and practices are essential. A key objective of the research was to highlight the need for drug testing services at festivals, providing attendees with a practical tool to ensure safer drug use.

Additionally, the study sought to stimulate open dialogue around drug use, fostering greater awareness and reducing misconceptions. By addressing stigma, the research emphasised that not all drug use is inherently problematic, advocating for a nuanced perspective that supports harm reduction initiatives. Finally, the study sought to legitimise harm reduction practices within the context of festival culture, underscoring their importance in protecting public health and well-being. This methodology ensures that the findings are both rigorous and context-sensitive, offering meaningful contributions to harm reduction research and policy advocacy.

## Results

### Welfare-focused harm reduction

Help and Support: Welfare Services One of the key themes that emerged in the study is the awareness and use of welfare services at festivals. The overwhelming majority, 82%, confirmed they knew whom to contact in the event of substance-related concerns. When asked about preferred points of contact, 34% named "Welfare," which rose to 54% when responses were expanded to include terms like "Welfare Tent" and "Welfare Workers." This points to a clear recognition of welfare services as essential at festivals.

However, despite this recognition, many participants reported that they had not encountered welfare tents or Ithink services prior to attending the festival. This highlights the need for greater visibility and accessibility of these services. Although 72% of respondents knew the location of the welfare tent, a notable proportion of attendees only became aware of these services during the festival itself. This finding suggests that more proactive efforts are needed to promote welfare services before and during events. A significant gap was also identified in participants' knowledge about broader substance use services. While many expressed a desire for more accessible harm reduction resources, 46% were unaware of existing programs. This highlights the necessity for a more structured and visible educational framework on available services, particularly regarding substance use. Further integration of welfare services and improved communication about their availability could help address this gap and encourage greater engagement.

Education and Understanding of Substances: Moving Beyond Fear-Based Approaches The understanding of drug use and education was another critical theme in the research. Participants rated their understanding of the UK drug classification system at an average of 6.7 out of 10, indicating a moderate level of awareness but also highlighting gaps in comprehensive education. More than half of the participants (58%) reported not receiving any formal drug education, and of those who had, 72% felt that the education was inadequate or failed to incorporate harm reduction principles.

One participant reflected on the shortcomings of traditional fear-based drug education: *"The demonisation of drugs stops people from having real conversations about why they use them and how to stay safe."* This sentiment aligns with broader critiques of current educational models, which often emphasise abstinence and the dangers of drug use without offering practical harm reduction strategies. The reliance on fear-based messaging and the stigmatisation of drug users prevents meaningful dialogue and the development of effective support systems.

The data also revealed that 90% of respondents rely on online resources for information about drugs. However, 52% of participants had never heard of harm reduction practices, underscoring a significant gap in public knowledge. These findings underscore the urgent need for targeted educational campaigns that critically address the complex interplay of stigma, social control, and criminalisation in shaping public perceptions of drug use. Rather than resorting to reductive moral panic narratives [38], such initiatives should be firmly grounded in evidence-based harm reduction strategies that enhance both public understanding and health outcomes [25]. By integrating and contextualising rich qualitative data and participant quotes, these campaigns can move beyond superficial portrayals to illuminate how stigma operates in diverse ways—undermining individuals’ willingness to access necessary services and reinforcing harmful societal stereotypes. In doing so, they can foster a more empathetic and informed dialogue on substance use, ultimately contributing to more effective public health interventions.

The issue of stigma was a recurring theme throughout the findings, particularly regarding substance use at festivals. Stigma can significantly impact individuals' willingness to seek help and access services. Some participants expressed hesitation in approaching welfare tents or seeking support due to concerns about judgment or negative perceptions. The concept of stigma was also evident in responses about the perceived risk of purchasing substances on-site versus bringing them from trusted sources. One participant stated, *"You don’t know what you’re getting at the festival, but it feels safer to bring it with you."* This response highlights how stigma surrounding drug use shapes risk perceptions, leading individuals to prioritise obtaining substances from familiar sources prior to attendance, despite the risks of arrest or expulsion, over purchasing from an unknown dealer within an event. In the absence of a drug-checking model, individuals may be unable or unwilling to engage with welfare or harm reduction services, particularly if these are perceived as judgmental. If such a model were available, it could facilitate engagement with these services, providing advice and information on substance risks and potentially mitigating harm.

Further analysis of stigma reveals that it not only shapes individual decisions but also impacts the overall effectiveness of harm reduction strategies. Participants indicated that a lack of open, non-judgmental spaces for discussion was a key barrier to engagement with welfare services. Ensuring that harm reduction services foster trust, maintain anonymity, and are framed as supportive rather than punitive could mitigate these challenges and encourage greater utilisation.

The concept of drug testing emerged as a pivotal issue in the study. The majority (74%) of respondents expressed a willingness to use a drug testing service if available at a festival. This suggests strong support for such services, which could reduce the risks associated with purchasing substances from unknown sources. However, a key barrier identified was concern over legal repercussions and potential law enforcement involvement. One participant noted, "Even if the testing service was there, I’d still be worried about the police."

This indicates that while drug testing services are broadly supported, fears of legal consequences may deter individuals from using them. To address this, ensuring that testing services are anonymous and operate independently of law enforcement could enhance uptake. Additionally, public education on the role of drug testing in harm reduction may help shift perceptions, moving away from viewing such services as an endorsement of drug use to recognising them as a vital safety measure.

A central concern among festival-goers regarding illicit substance use is the risk of consuming substances adulterated with harmful chemicals. As highlighted by Brunt [9], tailored approaches to testing different types of illicit drugs, such as ecstasy (MDMA), phenethylamines, and ketamine, are necessary given their widespread use in festival settings. These substances differ significantly from 'traditional' illicit drugs like cocaine and heroin, requiring different harm reduction considerations. The growing prevalence of "club drugs"—including stimulants, entactogens, and hallucinogens—reflects their use in festival environments, where they are often consumed to enhance the event experience [21].

The provision of drug-checking services is a key measure in reducing the risks associated with consuming adulterated or misidentified substances. One participant captured the anxieties surrounding drug content unpredictability: "Yes. They don't always work the same, so some pills are different. I worry what chemicals are being used and how it will affect my health." This statement reinforces the importance of providing reliable, harm reduction-focused testing services that address both legal and health concerns, ensuring that individuals have access to accurate information about the substances they consume.

The findings highlight both the effectiveness and the challenges of implementing drug-checking services at festivals. Evidence suggests that these services play a critical role in harm reduction by providing immediate, accurate information, encouraging safer consumption practices, and engaging individuals who may not typically seek healthcare support. However, barriers such as police presence, legal concerns, and social stigma continue to deter some festivalgoers from accessing these services. The fear of judgment both from authorities and peers further complicates uptake, underscoring the importance of framing drug-checking as a non-punitive, welfare-based intervention.

The discussion that follows will critically examine these findings within the broader context of harm reduction and drug policy. It will explore the implications for festival environments, considering how policy shifts such as decriminalisation and enhanced welfare measures could improve engagement with harm reduction services. Additionally, it will assess the role of policing and social control in shaping festivalgoers’ experiences, highlighting the need for evidence-based, compassionate approaches to drug use.

## Discussion

### Effectiveness of drug-checking services

The need for on-site drug-checking services at festivals is further supported by research indicating that these events see higher rates of drug use among attendees, particularly young people [26]. Drug-checking, which involves analysing substances for their chemical composition, allows users to make informed decisions, reducing the likelihood of adverse effects, such as overdoses or poisoning. On-site testing provides immediate, accessible information, an advantage over home-testing kits or amnesty bins, which can be less effective in preventing harm due to their lack of engagement and immediacy.

Studies, such as those by Measham [43], Valente et al. [66] have shown that drug-checking services can engage young adults who may not typically seek healthcare services, providing brief interventions (BIs) that effectively promote safer drug-use practices. These interventions not only inform users but also foster behaviour change [5]. Measham and Turnbull [42] note that after receiving test results, users are more likely to dispose of harmful substances or adjust their consumption, reducing the risk of drug-related harm. Unlike home-testing kits or amnesty bins, which do not provide real-time feedback or interaction, on-site drug-checking enables a direct dialogue with professionals, which enhances the effectiveness of harm reduction efforts. Participants in the study emphasised their trust in on-site services, with one noting, “I feel like they are there to help, not judge me. It’s safer when you know they’re not going to report you.” This shows that on-site drug-checking services not only inform users but also offer immediate support, making them more effective at reducing harm than passive alternatives.

### Concerns about police involvement and stigma

Despite the benefits of drug-checking services, concerns remain about the potential involvement of law enforcement. The study found that 74% of participants expressed apprehension about engaging with drug-testing services if they were associated with police presence or data collection. As one participant noted, "I would be worried about giving too much personal data, the police hanging around, worried I may be followed & arrested later on." This concern about privacy and legal implications is critical, as it directly affects users' willingness to engage with harm reduction services.

The stigma surrounding drug use and the fear of legal repercussions further complicate the uptake of drug-checking services. Participants expressed unease about the judgment they might face, with one remarking, "Stigma and judgment, having drugs confiscated…a Police Presence would make me feel uneasy, as I’d feel like I was under the eye of the police! (Unless they state no prosecution)." This highlights how stigma is not only external (from society or authorities) but also internalised, as some users fear being labeled as "reckless" or "irresponsible."

The study also reveals that participants are concerned about social stigma within the festival community. In environments where peer groups often define who is a "responsible user" versus a "reckless" one, the introduction of drug-checking services without clear safeguards against judgment can discourage engagement. As one participant shared, "I worry that others would see me at the booth and think I’m out of control or not being responsible." To address these concerns, the implementation of drug-checking services must be framed as anonymous and non-punitive, ensuring that festival-goers feel comfortable seeking assistance without fear of legal consequences or social stigma.

### Implications for harm reduction and policy

The findings from this study emphasise the importance of on-site drug-checking services in festival environments, particularly for reaching at-risk populations such as young people. By providing accurate and timely information about substances, these services play a crucial role in preventing harm, reducing overdose incidents, and promoting safer drug-use practices. However, the success of these services hinges on the trust and perception of festival-goers, which can be influenced by concerns about privacy, stigma, and police involvement.

To enhance the effectiveness of drug-checking services, it is essential to build trust with users, ensure anonymity, and mitigate any potential legal concerns. Policies should prioritise harm reduction by incorporating clear communication about the non-punitive nature of these services and fostering positive relationships between service providers and festival attendees. This approach aligns with the broader goal of creating safer, more informed festival environments where individuals can make educated choices about their health and well-being.

The research advocates for the continued integration of drug-checking services into festival settings, highlighting their role not only in reducing immediate harm but also in shaping long-term attitudes toward drug use and policy. By shifting the focus from criminalisation to education and harm reduction, these services can contribute to a broader cultural shift, fostering a more compassionate and evidence-based approach to substance use at festivals.

### Decriminalisation: reframing the narrative

The UK’s current approach to drug use at festivals largely revolves around criminalisation and punitive measures, which often lead to unintended harm and heightened risks for festivalgoers. Fear of legal consequences often discourages individuals from seeking essential medical help during critical moments, thus exacerbating the dangers associated with substance use. This section explores the harmful effects of criminalisation and advocates for a shift towards decriminalisation and welfare-based strategies. By adopting harm-reduction frameworks and fostering a supportive environment, festivals can not only enhance safety but also reduce stigma and encourage informed decision-making. Decriminalisation, combined with comprehensive welfare services, not only mitigates the risks linked with substance use but also fosters a culture of care and responsibility. This approach could serve as a model for public gatherings, underscoring the priority of health and well-being over punitive measures.

Under the Misuse of Drugs Act 1971, drugs in the UK are categorised into Class A, B, and C substances based on their perceived harm and potential for misuse. This legislation enforces strict penalties for the possession, distribution, and production of controlled substances, reinforcing a criminalisation approach that discourages individuals from seeking help due to fear of prosecution. This framework underscores the necessity for a shift toward welfare-based, harm reduction strategies, such as decriminalisation. Decriminalisation, which entails reducing or removing criminal penalties for certain drug-related activities, shifts the focus from punitive measures to public health and safety. Research shows that decriminalisation can help reduce drug-related harm by reducing stigma and legal consequences, and encouraging individuals to seek help [31]. In Portugal, where drug decriminalisation has been enacted, there have been significant reductions in drug-related deaths, HIV infections, and overall drug use [23].

Decriminalisation enables a more open dialogue about drug use, helping individuals access harm-reduction services without the fear of legal ramifications. This approach improves health outcomes and allows for more effective management of drug-related issues within communities [7]. By removing criminal penalties associated with drug possession, individuals are more inclined to engage with health services, seek information about safe drug use, and access addiction support if necessary. Welfare-based strategies focus on prioritising individuals’ health and well-being over punitive measures. These services include drug-checking, safe consumption spaces, and mental health and addiction support. At festivals, these approaches ensure that attendees can access medical care, safe spaces for recovery, and educational resources regarding substance use [49].

For example, drug-checking services enable festivalgoers to test their substances, making them aware of potential risks and helping them make informed choices [4, 53]. Such services reduce accidental overdoses and prevent the consumption of dangerous adulterants commonly found in street drugs. Safe consumption spaces also offer a controlled environment where users can consume substances under the supervision of trained professionals, further reducing the risk of harm [15, 18]. Additionally, welfare-based approaches reduce the stigma surrounding substance use by fostering open, non-judgmental conversations. This promotes individuals seeking help without the fear of social or legal repercussions, leading to better health outcomes and a more supportive community atmosphere.

### Microcosms of control: policing and criminalised behaviours at festivals

Festivals present a unique setting for exploring societal issues related to policing and criminalised behaviours. These events, characterised by large and diverse crowds, amplify the impacts of law enforcement practices and the criminalisation of drug use. Festivals act as temporary communities, providing a concentrated environment where the effects of these policies can be closely examined. The interactions between festivalgoers and the police or security personnel reveal valuable insights into the broader implications of criminalisation and the urgent need for harm-reduction strategies.

The increasing use of private policing and security at festivals raises concerns about the exercise of social control and the reinforcement of stigma against certain groups. Private security firms, operating with less oversight than public police forces, often prioritise risk management and liability over the welfare of the community. This can lead to practices that marginalise festivalgoers. Gould [22] suggests that police forces at festivals often act like a ‘Wild West’ posse, manipulating the law to suit their needs, from excessive force to over-policing certain communities. The issues arising from private policing reflect broader social concerns about the use of surveillance and police tactics that contribute to community unrest [28].

These policing practices are linked to the ideas of stigma and labelling as outlined by Cohen [12]. Certain groups, such as drug users, are often labelled as deviant or as "folk devils," and subject to increased scrutiny and punitive measures. This stigmatisation alienates individuals, hindering efforts at harm reduction. Participants in this study expressed fears about potential consequences for engaging with drug-testing services. For example, Paul noted, *“I have worries about engaging, feeling like there would be consequences like getting into trouble.”* Similarly, Jake voiced concerns about the *“legal ramifications of using drug-checking services.”*

Insights from festivalgoers demonstrate the pervasive anxiety about engaging with drug-checking services due to fear of legal consequences. Paul’s and Jake’s apprehensions highlight the psychological barriers that prevent individuals from using harm reduction services. These fears are rooted in the rigid drug laws and policing tactics employed at festivals. The anxiety surrounding potential legal repercussions discourages many from seeking help, increasing the risks associated with drug use.

Despite participants’ willingness to engage with drug-checking services, the fear of punitive actions from security and police prevents many from taking part. Nathan, for instance, worried that security might confiscate his substances, stating, *“I worry about clashing opinions from drug-testing workers and security who may want to take them off me and give punishment for them.”* Milly echoed this concern, noting, *“A police presence would make me feel uneasy, as I’d feel like I was under the eye of the police! (Unless they state no prosecution).”* Alfie also expressed discomfort with the possibility of law enforcement involvement, worrying that drugs might be confiscated. These participant insights underscore the pressing need to shift from punitive measures to welfare-based harm reduction approaches. Promoting drug-checking services in a safe and non-judgmental environment can mitigate the risks of drug use at festivals. Reducing the stigma and legal fears associated with these services will help ensure the safety and well-being of attendees, fostering a culture of care and responsibility.

## Conclusion

The findings of this study highlight the essential role of welfare services and harm reduction strategies in enhancing the safety and well-being of festivalgoers. A majority of participants expressed a clear preference for drug-checking services, demonstrating a willingness to engage with these services when offered in a supportive, non-punitive environment. Despite this willingness, significant gaps in education and awareness about harm reduction persist, signaling the need for comprehensive educational initiatives focused on substance use in schools, communities, and at events [55]. For harm reduction strategies to be effective, drug-testing services must be implemented at festivals with minimal police presence and clear communication about their non-prosecutorial nature. This will reduce the risks associated with drug use and encourage a more open, trusting engagement with these services.

The evidence further suggests that integrating harm reduction strategies—including drug testing, enhanced education, and welfare-based support services—can significantly mitigate the risks associated with substance use at festivals [34]. By equipping attendees with accurate information and fostering informed decision-making, festivals can become safer spaces that prioritise the well-being of participants. Drug-checking services, as part of these strategies, empower users by providing timely and accurate information about the substances they are consuming, reducing the likelihood of adverse health outcomes [3]. However, challenges such as stigma and legal concerns continue to hinder the widespread acceptance and success of these services. Despite these barriers, the benefits of implementing drug-checking services are evident, and their widespread adoption should be a priority within harm-reduction frameworks at festivals and similar events.

The UK's current drug enforcement strategy has been widely criticised for its ineffectiveness and disproportionate impact on marginalised communities. While drug-checking services currently operate under this framework, their potential is constrained by criminalisation and punitive measures that continue to stigmatise individuals and deter them from seeking harm-reduction services. The frequent use of stop-and-search tactics, which disproportionately target drug offences, exacerbates community tensions and undermines trust in law enforcement, without effectively reducing drug availability or related harms [68]. This highlights the limitations of a punitive approach, particularly with regard to its public health outcomes.

Given these issues, there is a clear need for reform to overcome the limitations of the current framework. Shifting towards decriminalisation or further legal reforms would eliminate the punitive barriers that discourage individuals from engaging with harm-reduction services like drug-checking. This would facilitate a more inclusive approach to care, ensuring that individuals can access necessary services without fear of prosecution. Legal reforms, combined with welfare-based approaches at festivals, could create environments where attendees feel safe and supported in seeking help. By prioritising health and safety over criminalisation, we can address the immediate risks associated with substance use while tackling the broader societal stigma that prevents individuals from accessing support [38].

This shift extends beyond improving festival safety. It serves as a potential model for public health reform in broader societal contexts. By embedding harm-reduction and welfare-based principles into event management and drug policy, we can reduce the stigma surrounding drug use, create supportive spaces, and foster a culture of care and responsibility. These reforms would improve public health and safety across diverse community settings, transforming the way we approach substance use. Ultimately, a fundamental shift in drug policy, one that prioritises harm reduction over punitive measures, is essential to ensure the well-being of individuals, enhance public health outcomes, and create safer, more supportive spaces for all.

## Data Availability

Data can be provided on request. I own and manage the data set.
